# MUC1 alters oncogenic events and transcription in human breast cancer cells

**DOI:** 10.1186/bcr1515

**Published:** 2006-07-17

**Authors:** Christine L Hattrup, Sandra J Gendler

**Affiliations:** 1 Mayo Clinic College of Medicine, Mayo Clinic Arizona, Scottsdale, AZ 85259, USA

## Abstract

**Introduction:**

MUC1 is an oncoprotein whose overexpression correlates with aggressiveness of tumors and poor survival of cancer patients. Many of the oncogenic effects of MUC1 are believed to occur through interaction of its cytoplasmic tail with signaling molecules. As expected for a protein with oncogenic functions, MUC1 is linked to regulation of proliferation, apoptosis, invasion, and transcription.

**Methods:**

To clarify the role of MUC1 in cancer, we transfected two breast cancer cell lines (MDA-MB-468 and BT-20) with small interfering (si)RNA directed against MUC1 and analyzed transcriptional responses and oncogenic events (proliferation, apoptosis and invasion).

**Results:**

Transcription of several genes was altered after transfection of MUC1 siRNA, including decreased *MAP2K1 *(MEK1), *JUN*, *PDGFA*, *CDC25A*, *VEGF *and *ITGAV *(integrin α_v_), and increased *TNF*, *RAF1*, and *MMP2*. Additional changes were seen at the protein level, such as increased expression of c-Myc, heightened phosphorylation of AKT, and decreased activation of MEK1/2 and ERK1/2. These were correlated with cellular events, as MUC1 siRNA in the MDA-MB-468 line decreased proliferation and invasion, and increased stress-induced apoptosis. Intriguingly, BT-20 cells displayed similar levels of apoptosis regardless of siRNA, and actually increased proliferation after MUC1 siRNA.

**Conclusion:**

These results further the growing knowledge of the role of MUC1 in transcription, and suggest that the regulation of MUC1 in breast cancer may be more complex than previously appreciated. The differences between these two cell lines emphasize the importance of understanding the context of cell-specific signaling events when analyzing the oncogenic functions of MUC1, and caution against generalizing the results of individual cell lines without adequate confirmation in intact biological systems.

## Introduction

MUC1 is the founding member of the mucin family: proteins characterized by heavy O-glycosylation centering around a variable number of tandem repeats that are rich in serine and threonine residues [1,2]. MUC1 is a transmembrane heterodimer with one subunit solely extracellular (MUC1-EX), and the other subunit composed of a short extracellular stem, a single transmembrane domain, and the cytoplasmic tail (together called the MUC1-CT). MUC1 possesses both pro- and anti-adhesive capacities, as the MUC1-EX provides binding sites for a variety of adhesion proteins, while its large size and extended structure prevents cell-cell contact [3-5].

Initially described as a tumor antigen overexpressed in >90% of breast cancers, MUC1 is now known to be an oncogene with roles in both tumor formation and progression [1,6]. Mouse studies have been integral to the current understanding of MUC1 in cancer. Muc1 knockout mice (Muc1^-/-^; MUC1 is human; Muc1 is mouse) show a reduction in tumorigenic phenotype when crossed onto mice overexpressing the Wnt-1 [7] or polyomavirus middle T antigen [8] oncogenes in the mammary gland. In contrast, MUC1 overexpression in the mammary gland drives tumor formation [9], indicating that MUC1 is a true oncogene.

Many of the oncogenic effects of MUC1 stem from its cytoplasmic tail, which binds to several proteins implicated in cancer, including c-Src [10,11] and the epidermal growth factor receptor (EGFR) family [12,13]. MUC1 stimulates mitogen activated protein kinase (MAPK) signaling through the extracellular signal regulated kinases (ERK1/2) [12,14]; this can occur through MUC1 association with Grb2 and son of sevenless to activate Ras [15]. ERK1/2 signaling is commonly stimulated by the Ras-Raf-MEK (MAPK and ERK kinase) cascade downstream of mitogens such as EGFR [16], and regulates transcription via factors like the activator protein-1 complex. Loss of MUC1 can reduce EGFR expression [17], providing another means of affecting MAPK signaling. Our results describe a novel mechanism by which MUC1 regulates the ERK1/2 pathway, through modulating transcription of the genes encoding MEK1, Raf-1, and c-Jun.

MUC1 expression correlates with increased survival in response to cytotoxic or oxidative agents [18-21], and can activate the phosphoinositol-3 kinase-AKT pathway as part of an anti-apoptotic response [18]. MUC1 has also recently been linked to transcription, as the MUC1-CT localizes to the nucleus [22] and affects transcription by β-catenin [22,23], FOXO3a [21], p53 [24], and estrogen receptor α [25]. However, there are indications that the role of MUC1 in oncogenesis is regulated by cell type and signaling context. For example, MUC1 can stimulate Fas-mediated apoptosis [26], while Muc1 is specifically down-regulated in c-neu-induced mammary tumors [27]. This report emphasizes the complexity of MUC1 signaling in breast cancer by contrasting results from two established breast cancer cell lines.

To understand MUC1 function in cells with high endogenous expression, that is, cells likely to have evolved with active MUC1 signaling, we used small interfering RNA (siRNA) to knock down MUC1 in MDA-MB-468 and BT-20 cells. We then analyzed transcription of 84 genes involved in cancer, as well as the effects upon cellular events linked to oncogenesis, such as apoptosis and proliferation. Though the cell lines show some similarity in transcriptional alterations after transfection with MUC1 siRNA, their phenotypes are quite dissimilar: MDA-MB-468 increases apoptosis and reduces proliferation and invasion, while BT-20 proliferates more rapidly after loss of MUC1. This last may reflect the striking amount of active AKT in BT-20; AKT activity is increased in both cell lines after MUC1 siRNA, which agrees with a previous study of MUC1 siRNA [21], but disagrees with results from 3Y1 fibroblasts [18]. Recent studies have emphasized the complex and context-specific regulation of even such classic oncogenes as AKT [28]. The differences between the two breast cancer cell lines in this study suggest that MUC1 oncogenic functions are also subject to cell-specific regulation, and stress the need for understanding the cellular signaling context when interpreting results.

## Materials and methods

### Cell culture and siRNA transfection

MDA-MB-468 and BT-20 cells (American Type Culture Collection) were cultured in Dulbecco's modified Eagle's medium (Invitrogen, Carlsbad, CA, USA) plus 10% fetal calf serum, 1% Glutamax (Invitrogen) and 1% penicillin/streptomycin. Stable cell lines (468.Neo and 468.MUC1Δ8) were selected with 0.5 mg/ml G418. For epidermal growth factor (EGF) stimulation, MDA-MB-468 cells were serum-starved overnight and treated for 10 minutes at 37°C with 100 ng/ml EGF. Transient siRNA transfection was performed with Lipofectamine2000 (Invitrogen) and 100 nM siRNA oligonucleotides. The commercially available siRNA constructs (all from Dharmacon, Lafayette, CO, USA) were scrambled (siCONTROL non-targeting siRNA #1), or directed against firefly luciferase (siCONTROL non-targeting siRNA #2) or MUC1 (siGENOME smartpool). The independent oligonucleotides designed in our laboratory target sequences beginning at MUC1 codons 882 and 956, and have been described previously [29]. The scrambled siRNA construct was used only in BT-20 cells as it causes a non-specific knockdown of MUC1 in the MDA-MB-468 line.

### Cloning of MUC1 WT vector and stable transfection

Two silent mutations (G891A and T894C) were introduced into the MUC1 cDNA (called MUC1Δ8) to make it resistant to the 882 siRNA that targets that region of the mRNA. The mutant cDNA was cloned into the pLNCX.1 vector with neomycin resistance (gift of Joseph Loftus, Mayo Clinic, Arizona, USA). Stable transfection was performed with Lipofectamine2000; cells were selected beginning 24 hours post-transfection and maintained as a polyclonal population.

### Western blots and antibodies

Cells were lysed in buffer (20 mM HEPES pH 8.0, 150 mM sodium chloride, 1% Triton X-100, 2 mM EDTA) with commercial protease (Complete inhibitor cocktail, Roche, Pleasanton, CA, USA) and phosphatase inhibitors (10 mM sodium fluoride, 2 mM sodium vanadate, 50 μM ammonium molybdate). Protein concentration was determined by BCA (Pierce, Rockford, IL, USA); 50 μg of lysate were loaded on SDS-PAGE gels for each experiment, except for the pMEK1/2 blot in MDA-MB-468, where 150 μg were used. Non-commercial antibodies used were: BC2, a mouse monoclonal to the MUC1-EX (gift of Dr McGuckin, Queensland University, Queensland, Australia), and CT2, an Armenian hamster monoclonal to the MUC1-CT developed in our lab [12]. Antibodies to pMEK1/2, MEK1/2, ERK1/2, Myc, pAKT, AKT, β-tubulin (all Cell Signaling, Danvers, MA, USA), β-actin and dpERK1/2 (both Sigma, St. Louis, MO, USA) were used according to manufacturers' recommendations. All antibodies except β-actin (1:2,500) and dpERK1/2 (1:10,000) were used at 1:1,000 dilution for western blots. Flow cytometric analysis of MUC1 was done with HMPV-FITC, which recognizes the core peptide of the MUC1-EX tandem repeats (Pharmingen, San Diego, CA, USA). Bromodeoxyuridine (BrdU) staining was performed with a fluorescently conjugated antibody to BrdU (BrdU-PE, BD Biosciences, San Diego, CA, USA) as described below. Densitometry was performed using the public domain ImageJ program (developed at the NIH and available at [30]. Each band was measured in three places; the results were averaged and normalized to tubulin to control for loading.

### Transwell invasion assays

Cells were serum-starved beginning 24 hours post-siRNA transfection. Cells were re-plated in serum-free medium 48 hours post-transfection at 50,000 cells per insert (sized for 24-well plates), with serum-containing medium in the bottom of the growth well as an attractant. Transwell inserts (BD Biosciences) pre-coated with laminin, fibronectin, collagen IV, or control (no matrix) were used, and cells were permitted to invade for 48 hours. At this point (96 hours post-transfection), visual inspection of the growth wells confirmed that negligible numbers of cells went through to the bottom of the plate. Non-invaded cells were swabbed from the tops of half of the inserts ('samples', containing only invaded cells), and retained in the others ('controls', all cells). Inserts were stained for 10 minutes with crystal violet (0.5% in 20% methanol) and washed with water. Membranes were cut out and destained for 10 minutes in 10% acetic acid in a 96-well plate; membranes were removed and absorbance was read at 570 nm. Percent invasion is defined as (absorbance of samples/absorbance of controls) × 100.

### [^3^H]Thymidine incorporation assays

Cells were re-plated in quadruplicate 24 hours post-siRNA transfection at 15,000 cells/well (96-well plate) with [^3^H]thymidine (1 μCi/well), then incubated in normal conditions for 24 hours. At this time (48 hours post-siRNA transfection) excess radioactivity was washed off and the cells were harvested and read on a TopCount plate reader. Statistical analysis was performed using JMP 5.1.2 software (SAS Institute, Inc., Cary, NC, USA); the student's *t *test was used to determine *p *values and significance was confirmed with Wilcoxon rank sum and Pearson chi squared analyses.

### BrdU incorporation

BrdU (50 μM) was given to cells 48 hours post-siRNA transfection and permitted to incorporate for 1.5 hours. Cells were then washed with PBS, trypsinized, and washed again. BrdU staining was performed according to an adaptation of the manufacturer's protocol: cells were re-suspended in PBS, mixed 1:1 with -20°C neat ethanol, and incubated 1 hour at -20°C to fix. Fixed cells were then washed gently and denatured in 2 M HCl for 20 minutes at room temperature. Following washing and 2 minute's incubation with 0.1 M Tris to neutralize the acid, cells were re-suspended in FACS buffer (0.5% fetal calf serum in PBS) and stained with Phycoerythrin (PE)-conjugated anti-BrdU according to the manufacturer's protocol for flow cytometry analysis on a FACScan instrument.

### Apoptosis and trypan blue staining

Apoptosis was measured using a kit (BD Biosciences) containing propidium iodide (PI) and FITC-conjugated annexin V. Cells were stained according to the manufacturer's protocol and the level of apoptosis determined by flow cytometry. Quadrants are: early apoptosis (annexin V^+^/PI^-^, lower right) late apoptosis (annexin V^+^/PI^+^, upper right) and non-apoptotic cell death (annexin V^-^/PI^+^, upper left). Treatments for the stress panel were: no treatment (control); DMSO as a control for celecoxib; 20 mM celecoxib, brand name Celebrex™ (dissolved in DMSO) [31]; 0.2 mM H_2_O_2 _[32]; or 1 mg/mL G418 (Pfizer, New York, NY, USA).

### Real-time PCR arrays

Transcriptional analysis using Cancer PathwayFinder RT^2 ^profiler arrays (SuperArray, Frederick, MD, USA) was performed according to the manufacturer's protocol. Briefly, total RNA was isolated using an RNeasy extraction kit (Qiagen, Valencia, CA, USA); 1 μg of RNA was reverse transcribed with the cDNA synthesis kit (SuperArray) and cDNA was subjected to real-time PCR using SYBR green to detect product. Arrays were performed independently at least twice for each cell line; all PCR products were checked on agarose gels. Values were obtained for the threshold cycle (C_t_) for each gene and normalized using the average of four housekeeping genes on the same array (*HPRT1*, *RPL13A*, *GAPD*, *ACTB*). C_t _values for housekeeping genes and a dilution series of *ACTB *were monitored for consistency between arrays. Change (ΔC_t_) between MUC1 siRNA and control siRNA was found by:

ΔC_t _= C_t(MUC1 siRNA) _- C_t(control siRNA)_

and fold change by:

Fold change = 2^(-ΔCt)^

Values are given as fold change; only genes showing two-fold or greater change were considered. Both luciferase and scrambled siRNA controls were used in BT-20; only genes showing consistent alteration with both controls were included in the results reported here. The scrambled siRNA could be not used in MDA-MB-468 as these cells decrease MUC1 expression in response to this construct.

## Results

### siRNA transfection decreases MUC1 expression in breast cancer cell lines

**Figure 1:**
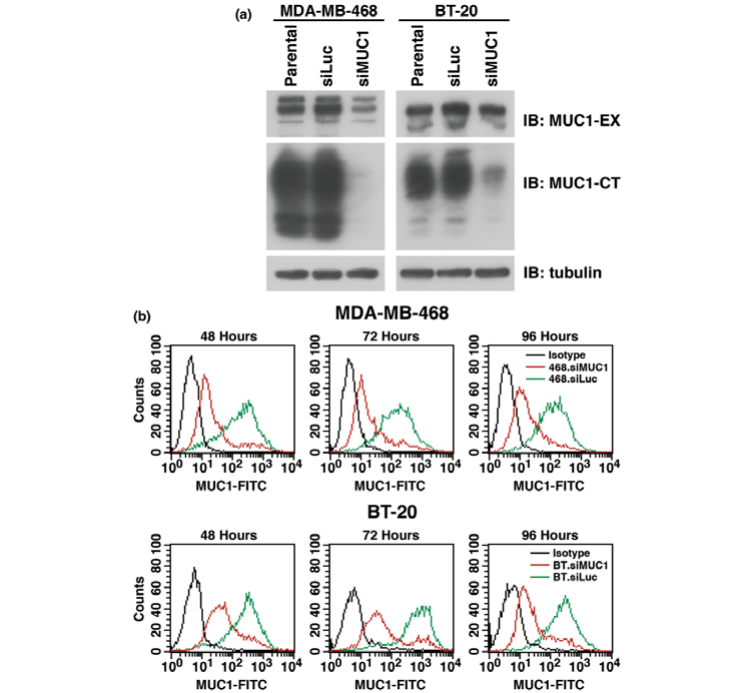
Small interfering RNA (siRNA) decreases MUC1 levels in breast cancer cells. **(a) **Western blots (IB) of the MUC1 cytoplasmic tail (MUC1-CT) and extracellular domain (MUC1-EX) in untreated (parental), or luciferase siRNA treated (siLuc) or MUC1 siRNA (siMUC1) treated MDA-MB-468 and BT-20 cells. Lysates were analyzed 48 hours post-transfection; tubulin is shown as a loading control. **(b) **Flow cytometry analysis of the MUC1 extracellular domain in unpermeabilized, siRNA-treated MDA-MB-468 and BT-20 lines. Cells were analyzed at 24 hour intervals beginning 48 hours post-transfection. The black line represents isotype control, the green luciferase siRNA (siLuc), and the red MUC1 siRNA (siMUC1).

Two human breast cancer cell lines, MDA-MB-468 and BT-20, were transiently transfected with a pool of four siRNA oligonucleotides directed against the MUC1 mRNA (468.siMUC1 and BT.siMUC1), or a control oligonucleotide directed against luciferase (468.siLuc and BT.siLuc). Both cell lines express high levels of MUC1, making them promising targets for this analysis. Western blots (Figure 1a) show successful knockdown of both the extracellular domain and cytoplasmic tail fragments of MUC1; luciferase siRNA does not substantially change the level of MUC1 compared to parental cells. 468.siMUC1 show a substantial decrease in the amount of MUC1-CT, while BT.siMUC1 show slightly less knockdown of MUC1-CT. Both MDA-MB-468 and BT-20 display a less dramatic decrease of MUC1 extracellular domain compared to MUC1-CT (Figure 1a); this likely represents protein synthesized prior to transfection, and may reflect differences in the turnover rates of the two subunits.

Analysis of the MUC1 extracellular domain by flow cytometry confirms that both cell lines substantially decrease MUC1 expression after siRNA (Figure 1b). By flow cytometry, 468.siMUC1 averaged 75% knockdown of MUC1 compared to 468.siLuc; and BT.siMUC1 averaged 50% knockdown relative to BT.siLuc. These effects could be titrated with the concentration of siRNA, were seen as early as 24 hours post-transfection (data not shown) and lasted to at least 96 h post-transfection (Figure 1b). All experiments were conducted within 48 to 96 hours after siRNA transfection. Similar results were obtained using two independent oligonucleotides designed in our lab (data not shown), designated '882' and '956' for the initial codon recognized by each.

### Transcriptional changes are seen after MUC1 siRNA

**Figure 2:**
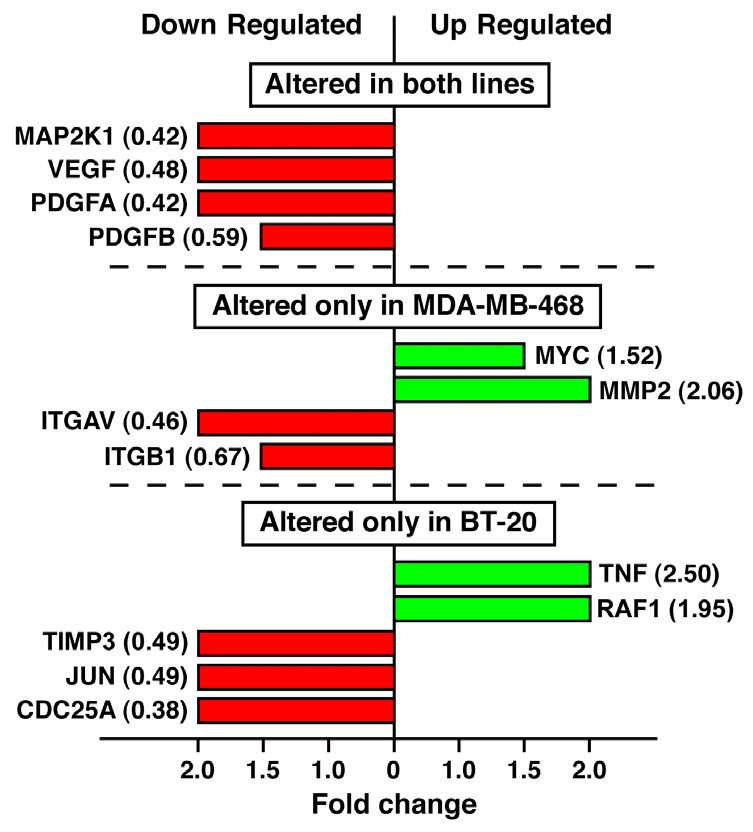
Transcription of genes involved in cancer is altered in response to MUC1 small interfering RNA (siRNA). Genes whose transcription was altered by at least two-fold in MUC1 siRNA-treated cells compared to control siRNA are shown. Green bars represent genes whose transcription was up-regulated; red bars are genes whose transcription was down-regulated after MUC1 siRNA. The average fold change is shown in parentheses after the gene name; for genes altered in both MDA-MB-468 and BT-20, these values reflect the average from both cell lines.

**Figure 3:**
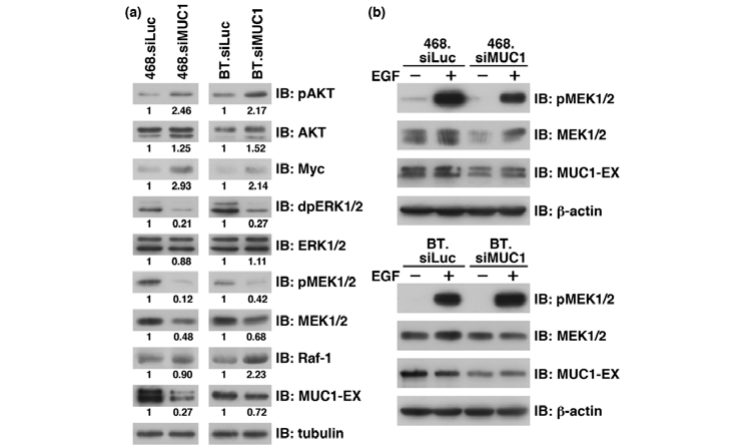
Western blot analysis confirms changes in protein levels after MUC1 small interfering RNA (siRNA). **(a) **Whole cell lysates were analyzed for the following proteins: MUC1 extracellular subunit (MUC1-EX), c-Myc, total and active MAPK and ERK kinase (MEK)1/2 (MEK1/2, pMEK1/2), total and active extracellular signal regulated kinase (ERK)1/2 (ERK1/2, dpERK1/2), total and active AKT (AKT, pAKT), and tubulin as a loading control. Densitometry is shown for each blot, based on three independent measurements per band, normalized to tubulin; results are shown relative to luciferase siRNA (siLuc) for each cell line. Optimal exposures for comparing luciferase siRNA and MUC1 siRNA-treated cells from the same parental line are shown. **(b) **Lysates from epidermal growth factor (EGF)-stimulated MDA-MB-468 cells were blotted for MEK1/2, pMEK1/2, MUC1-EX, and actin (for loading).

Recent work indicates that MUC1 may affect transcription both directly via interaction with transcription factors and indirectly (for example, through modulating signaling). To study the effects of MUC1 knockdown in breast cancer cell lines, real-time PCR arrays were used to analyze transcription of 84 genes implicated in cancer. Only genes with greater than two-fold change were considered. Three genes (*MAP2K1*, *VEGF*, *PDGFA*) were altered two-fold or more after MUC1 siRNA in both MDA-MB-468 and BT-20 cells (Figure 2); two genes (*ITGAV*, *MMP2*) changed only in 468.siMUC1; and five genes (*TIMP3*, *RAF1*, *JUN*, *TNF*, *CDC25A*) only in BT.siMUC1. This list represents all genes affected greater than two-fold after MUC1 siRNA, rather than a select group. Three genes whose transcription was changed by less than two-fold are shown, two of which (*PDGFB *and *ITGB1*) are listed because they relate closely to genes altered by two-fold (*PDGFA *and *ITGAV*). The third, *MYC*, is included because western blots confirmed a substantial change at the protein level (Figure 3a) that may reflect both transcriptional and post-transcriptional regulation.

Interestingly, transcription of *MAP2K1 *was decreased in both cell lines after MUC1 siRNA. This gene encodes MEK1, one of the primary regulators of the ERK1/2 MAPK pathway [33], a network that has been linked several times to MUC1 [12,34-36]. We examined MEK1 and MEK2 levels by western blot to confirm decreased protein in MUC1 siRNA-treated cells (Figure 3a), and found that not only were total MEK1/2 levels lower in 468.siMUC1 and BT.siMUC1 compared to controls (0.48 and 0.68 relative to siLuc, respectively), but so were the basal amounts of active (phosphorylated) MEK1/2 (pMEK1/2; 0.12 and 0.42 relative to siLuc, respectively). Both 468.siMUC1 and BT.siMUC1 also showed reduced activation of ERK1/2 (dpERK1/2; 0.21 and 0.27 relative to siLuc, respectively), as would be expected with diminished signaling through MEK1/2; total ERK1/2 levels remain unchanged.

As both lines have high levels of EGFR and thus activate the MEK-ERK cascade intensely when stimulated with EGF [37], siRNA-transfected cells were treated with EGF. Notably, MUC1 siRNA impairs this important oncogenic pathway in MDA-MB-468 cells, as 468.siMUC1 display less pMEK1/2 in response to EGF than do 468.siLuc (Figure 3b). Interestingly, EGF treatment of BT-20 cells results in slightly higher pMEK1/2 levels in BT.siMUC1 compared to BT.siLuc. Though this result seems paradoxical in light of decreased *MAP2K1 *transcription in BT.siMUC1, it likely results from differential functions of Raf isoforms in combination with the increased *RAF1 *transcription (Figure 2) and protein level (Figure 3a) in these cells. Specifically, B-Raf is thought to be the main activator of MEK under normal conditions; Raf-1 activates MEK in response to stimulus [38]. Thus, it appears that basal pMEK1/2 levels are not greatly affected by Raf-1 overexpression in BT.siMUC1 cells, likely because MEK is regulated primarily by B-Raf under normal growth conditions. In contrast, when the cells are stimulated (EGF), increased Raf-1 levels in BT.siMUC1 leads to heightened pMEK1/2 (Figure 3b).

### MUC1 siRNA increases apoptosis in MDA-MB-468 but not BT-20

**Figure 4:**
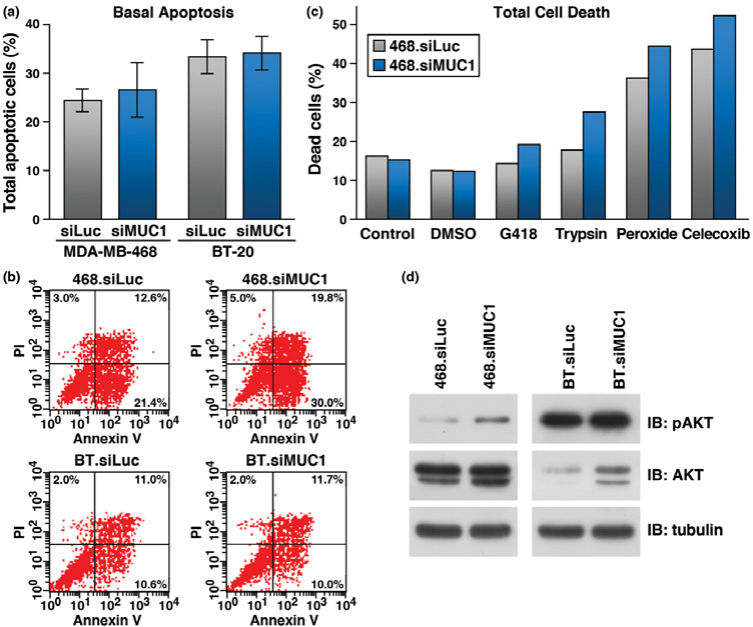
MUC1 small interfering RNA (siRNA) affects cell death in response to stress in MDA-MB-468, but not BT-20 cells. **(a) **Basal apoptosis is shown as the combined total of early (propidium iodide (PI)^-^/annexin V^+^) and late (PI^+^/annexin V^+^) apoptotic populations. Results reflect the averages of three independent experiments. **(b) **Apoptosis after trypsinization 24 hours post-transfection. Numbers in the quadrants reflect the percentage of cells in each. Representative flow cytometry results are shown for MDA-MB-468 and BT-20 cells transfected with siRNA. **(c) **Total cell death (sum of non-apoptotic cell death (PI^+^/annexin V^-^), and early and late apoptosis) is shown for MDA-MB-468 cells in response to a panel of cellular stresses (G418, trypsin, peroxide, celecoxib) and controls (control, DMSO). Representative results from three independent experiments are shown. **(d) **Western blots (IB) for total and active AKT (AKT, pAKT) and tubulin loading control, performed on whole cell lysates from siRNA transfected cells. The same exposure is shown for both cell lines to show the relative levels of protein between lines, rather than optimal exposure times for comparison within a single cell line (shown in Figure 3a).

We next examined whether MUC1 knockdown and its associated transcriptional alterations would affect overall cellular events. As several of the genes shown in Figure 2 are important in regulating proliferation and survival, and because of the recently described role of MUC1 in modulating apoptosis in response to cellular stresses [20,21,24], we first analyzed whether MUC1 siRNA would alter apoptosis in these lines. Although there was no change in basal apoptosis in either line (Figure 4a), we observed that the cell lines responded differently when trypsinized for re-plating 24 hours after transfection (Figure 4b). Interestingly, 468.siMUC1 cells show greater apoptosis after trypsinization than do 468.siLuc (49.8% versus 34.0%, respectively), while BT-20 cells from both siRNA treatments display similar levels of apoptosis (around 22%).

To examine whether this phenomenon is specific to trypsin treatment or part of a general stress response involving MUC1, we subjected cells to a panel of stresses and measured cell death. In agreement with the patterns seen with trypsinization, BT.siLuc and BT.siMUC1 respond similarly to all treatments (data not shown), while 468.siMUC1 die more readily than 468.siLuc in response to trypsin, G418, hydrogen peroxide, or celecoxib, a chemotherapeutic that targets the cyclooxygenase-2 (COX-2) pathway (Figure 4c); these data were confirmed with two independent siRNA constructs (data not shown).

Like the MAPK pathway, AKT signaling has been linked to MUC1 in cancer. Although transcription of *AKT *was not altered in MUC1 siRNA-treated cells, the results of our apoptosis studies prompted us to investigate levels of AKT further. As expected, the total AKT protein level is not greatly changed after MUC1 siRNA in either cell line, though the active form (pAKT) is increased in both 468.siMUC1 and BT.siMUC1 compared to controls (Figure 3a). This result disagrees with MUC1 activation of the AKT pathway in rat 3Y1 cells [18], and may reflect regulation more appropriate to breast cancer cells; this is supported by activation of AKT in response to MUC1 siRNA in other lines [21]. In addition, there is a striking difference in the relative amounts of AKT and pAKT in the two cell lines (Figure 4d). When lysates from both lines are exposed to film for the same length of time (overexposure masks the differences between BT.siLuc and BT.siMUC1 that are apparent in Figure 3a), it is clear that pAKT levels are much higher in BT-20 than in MDA-MB-468, despite lower total AKT expression. This difference in AKT activation between MDA-MB-468 and BT-20 likely contributes to the disparity in their sensitivity to the increased apoptosis expected with loss of MUC1.

### MUC1 siRNA alters proliferation and invasion

**Figure 5:**
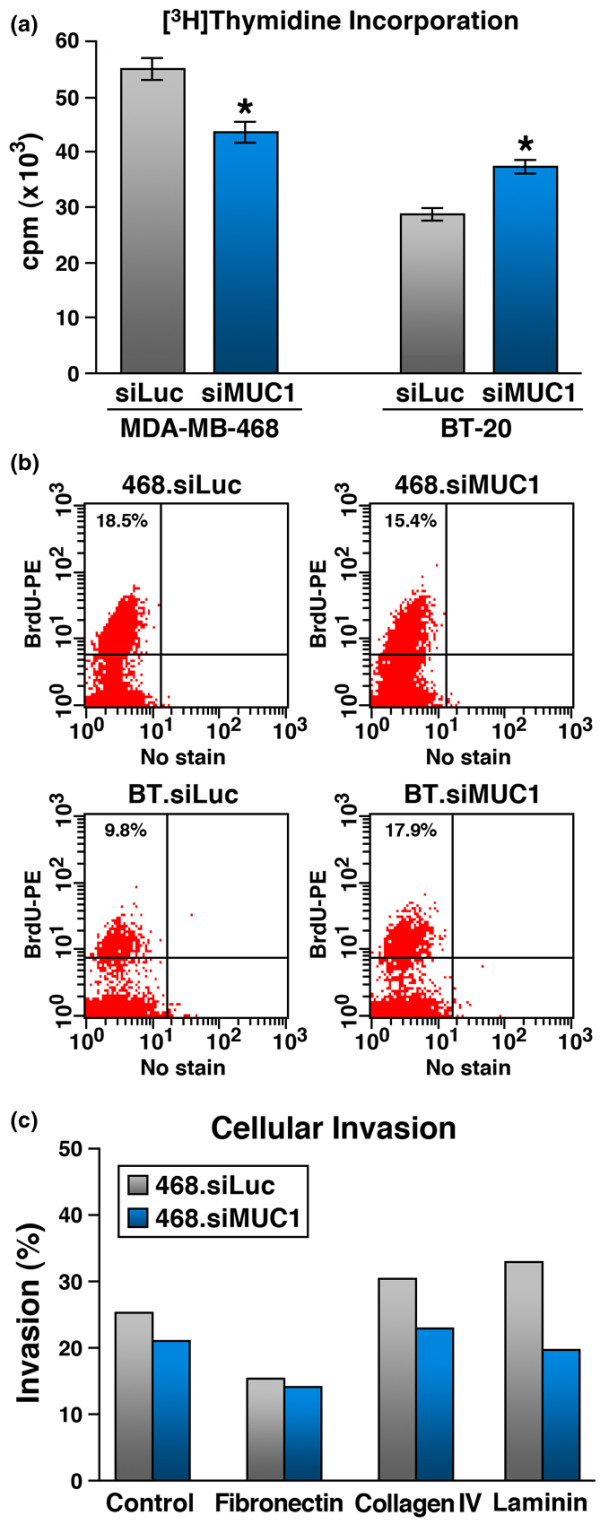
Proliferation is altered by MUC1 small interfering RNA (siRNA) in both cell lines. **(a) **[^3^H]Thymidine incorporation is shown by the level of radioactivity (cpm) emitted from cells after 24 hours incubation with radionucleotide. Results reflect the average of three independent experiments. *p < 0.006 compared to luciferase siRNA (siLuc) treated cells of the same line (that is, 468.siLuc or BT.siLuc). **(b) **Flow cytometric analysis of bromodeoxyuridine (BrdU) incorporation. siRNA-transfected cells were incubated with BrdU for 1.5 hours, and stained with a Phycoerythrin PE-conjugated antibody against BrdU. A representative experiment is shown. **(c) **Invasion of MDA-MB-468 cells in transwells plated with a panel of extracellular matrix proteins or no matrix (control). Representative results are shown as percent invasion, calculated as: number of invaded cells/total number of cells × 100.

As MUC1 is involved in apoptosis, we next analyzed its effects on proliferation. BrdU and [^3^H]thymidine incorporation were used to analyze proliferation after MUC1 siRNA. 468.siMUC1 cells show a significant decrease in [^3^H]thymidine incorporation compared to 468.siLuc, while intriguingly, BT.siMUC1 cells show a significant increase in proliferation (Figure 5a). Growth curves mirror these results, as do experiments with the two independent MUC1 siRNA oligonucleotides (data not shown). Note that these assays require trypsinizing cells 24 hours post-transfection; therefore, the results in the MDA-MB-468 line could stem from the changes in apoptosis described in the previous section, rather than a true effect on proliferation. To control for this, we incubated non-trypsinized, siRNA-transfected cells at similar confluence with BrdU to measure incorporation. The 'clumped' profile of cells (contrast to Figure 4b) is likely a result of the acid denaturation (recommended by the antibody manufacturer), as it occurs uniformly in these experiments. BrdU incorporation (Figure 5b) confirms that the [^3^H]thymidine results are not solely due to alterations in apoptosis, as 468.siMUC1 cells incorporate less BrdU than 468.siLuc; once again, BT.siMUC1 cells show increased proliferation over BT.siLuc.

Given the role of MUC1 in adhesion, we examined whether MUC1 siRNA affects cellular invasion. In transwell assays, BT-20 cells invaded poorly, regardless of the siRNA used (data not shown). However, MDA-MB-468 cells invade more readily, and were analyzed on a panel of three different extracellular matrix proteins. Interestingly, 468.siMUC1 cells display somewhat decreased invasion on collagen IV, laminin, and fibronectin matrices, and on a no-matrix control (Figure 5c), which is in agreement with the trend towards decreased metastasis observed in Muc1^-/- ^× MMTV-PyV MT mice [8].

### Transfection of MUC1 rescues the 468.siMUC1 phenotype

**Figure 6:**
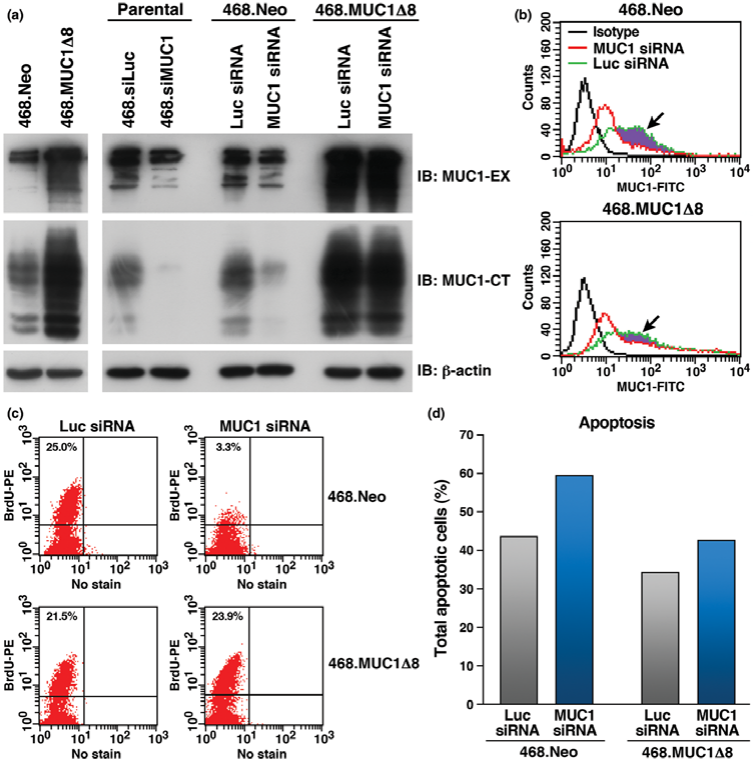
The MUC1 interfering RNA (siRNA) phenotype is rescued by stable transfection with MUC1. **(a) **MDA-MB-468 cells transfected with a full-length, siRNA-resistant MUC1 construct (468.MUC1Δ8) or empty vector (468.Neo); lysates were blotted for MUC1 extracellular (MUC1-EX) and cytoplasmic (MUC1-CT) domains, with actin as a loading control. The left panel shows untransfected cells; the right shows siRNA treatment of the stable transfectants and parental cells. **(b) **siRNA-transfected 468.Neo and 468.MUC1Δ8 cells were stained for expression of MUC1-EX 48 hours post-transfection and analyzed by flow cytometry. The black line represents isotype control, the green luciferase (Luc) siRNA, and the red MUC1 siRNA. Arrows point out the shaded regions that reflect the level of knockdown in each cell line. **(c) **Flow cytometric analysis of bromodeoxyuridine (BrdU) incorporation after 1.5 hours incubation with siRNA transfected cells. The number shown in each upper left quadrant reflects the percentage of BrdU-positive cells. A representative experiment is shown. **(d) **Total apoptosis is shown as the sum of early (propidium iodide (PI)^-^/annexin V^+^) and late (PI^+^/annexin V^+^) apoptotic populations for 468.Neo and 468.MUC1Δ8 cells trypsinized 24 hours post-transfection. A representative example is shown.

To determine if the above effects are specific to MUC1, we created stable transfectants of the MDA-MB-468 line using empty vector (468.Neo) or a full-length MUC1 construct (468.MUC1Δ8) that is resistant to one of the independent MUC1-directed oligonucleotides ('882'). These cells were maintained in G418-containing medium to retain transgene selection. As expected, 468.MUC1Δ8 cells show higher levels of both the MUC1 extracellular domain and the MUC1-CT than do 468.Neo (Figure 6a). Note that 468.Neo have MUC1 expression comparable to parental MDA-MB-468; the exposures in Figure 6a are lighter than those in Figure 1a, in order to clearly show the relative levels of MUC1 in the stable transfectants. After MUC1 siRNA, 468.MUC1Δ8 lose some MUC1 (likely endogenous protein, which is not siRNA-resistant) but retain high-level expression, while 468.Neo show a decrease in MUC1 levels similar to parental 468.siMUC1 cells (Figures 6a,b). The difference in the amount of MUC1 knockdown between 468.Neo and 468.MUC1Δ8 is highlighted by the purple shading in Figure 6b.

BrdU incorporation (Figure 6c) indicates that 468.Neo show decreased nucleotide incorporation after MUC1 siRNA compared to control (3.3% versus 25.0%, respectively); this is not seen in 468.MUC1Δ8 cells, which show similar levels of BrdU incorporation regardless of the siRNA used (21.5% for luciferase, 23.9% for MUC1). 468.Neo cells display a more dramatic decrease in BrdU incorporation after MUC1 siRNA than what is seen in parental 468.siMUC1 cells, which may reflect the additional stress of being maintained in G418-containing medium. Similarly, analysis of apoptosis in trypsinized cells indicates that the increased apoptosis seen in parental 468.siMUC1 cells is also present in the 468.Neo line after MUC1 siRNA (Figure 6d; 43.6% in control versus 59.6% in MUC1 siRNA). However, in 468.MUC1Δ8 cells, the level of apoptosis after luciferase siRNA (34.1%) is lower than that in 468.Neo cells; MUC1 siRNA increases the amount of apoptosis slightly (42.8%), restoring it to a level similar to that seen in luciferase siRNA-treated 468.Neo cells. Together, these studies suggest that the above-described results are specific to MUC1, as stable transfection of an siRNA-resistant MUC1 rescues the phenotype seen in 468.siMUC1 cells.

## Discussion

This report describes both the transcriptional alterations seen after transfection with MUC1 siRNA in human breast cancer cells and the effects on events such as apoptosis and proliferation. The two cell lines used (MDA-MB-468 and BT-20) were chosen for high expression of MUC1 and a substantial (50% to 75%), consistent decrease in MUC1 expression after siRNA. Both lines have epithelial morphology, form tumors slowly in nude mice [37], have mutant p53 [39,40], express EGFR [37], and lack estrogen receptor α [41]. One striking difference between these lines, however, is their response to MUC1 siRNA. MDA-MB-468 cells behave as expected for loss of an oncogene: MUC1 siRNA correlates with increased apoptosis in response to stress, decreased proliferation, and reduced invasion. In contrast, BT.siMUC1 cells proliferate more rapidly than BT.siLuc cells with little effect on apoptosis.

Much of the phenotype of these cells can be understood in light of protein levels and transcriptional activity after MUC1 siRNA. As mentioned, both MDA-MB-468 and BT-20 display increased pAKT after MUC1 siRNA, but the ratio of active to total AKT is considerably higher in the BT-20 line, which may help these cells resist the increased apoptosis expected with loss of MUC1. Myc levels are also higher in both cell lines after MUC1 siRNA, although the ability of Myc to promote proliferation and apoptosis in different cellular contexts [42] complicates the interpretation of this finding.

Both cell lines show reduced transcription of *VEGF*, *PDGFA*, *PDGFB*, and *MAP2K1 *(MEK1) after MUC1 siRNA. The genes encoding vascular endothelial growth factor (VEGF) and the A and B chains of platelet-derived growth factor (PDGF-A and PDGF-B) are interesting as these proteins have been heavily implicated in angiogenesis, suggesting a novel function for MUC1 in regulating this process. Vascular endothelial growth factor expression in cancer is linked to tumor growth and metastasis [43,44]; platelet-derived growth factor is also angiogenic, but has an additional role in stimulating desmoplasia [45]. Reduced transcription of these genes after MUC1 siRNA suggests that MUC1 may foster angiogenesis and stromal proliferation, although this must be confirmed in a more appropriate model system.

Decreased *MAP2K1 *(MEK1) transcription after MUC1 siRNA provides a novel mechanism by which MUC1 can affect the ERK1/2 MAPK pathway. MUC1 has often been linked to the Ras-Raf-MEK-ERK cascade [12,34-36,46], and at least two mechanisms by which MUC1 can alter MAPK signaling have been described: MUC1 interaction with and phosphorylation by the EGFR family [12,13], and MUC1 binding to the Grb2/Sos complex that activates Ras [46]. Reduction of MEK1 levels after MUC1 siRNA agrees with the role of MUC1 in strengthening MAPK signaling, and indicates that MUC1 can regulate both the transcription and activity of members of this pathway.

Two additional MAPK pathway members are altered specifically in BT.siMUC1, with no corresponding change in 468.siMUC1 cells. These genes are *RAF1 *and *JUN *which are increased and decreased, respectively, after MUC1 siRNA. Raf-1 and c-Jun both function outside of the ERK1/2 MAPK pathway, which may explain the seeming paradox of increased *RAF1 *transcription with simultaneous decreases in *MAP2K1 *and *JUN*. Specifically, Raf-1 can inhibit ASK1 (apoptosis signal-regulated kinase 1) upstream of p38 and JNK (Jun N-terminal kinase) [38]. ASK1 phosphorylates JNK in response to stress, resulting in activation of c-Jun and stimulation of apoptosis [47], indicating that the coordinate up-regulation of *RAF1 *and down-regulation of *JUN *may provide a potent anti-apoptotic effect in BT.siMUC1.

Regulation of life and death is also a hallmark of the *CDC25A *and *TNF *gene products. CDC25A is a phosphatase that stimulates cell cycle progression [48], thus the effects of its decrease in BT.siMUC1 are unclear in light of the increased proliferation of these cells. However, the CDC25 proteins (A, B, and C) were recently shown to have greater functional overlap than was previously thought [49], suggesting that the other two isoforms may compensate for reduced CDC25A levels. *TNF *encodes tumor necrosis factor (TNF)α, known for its potent, cell type-specific control of life and death. In tumor cells, TNFα expression can promote proliferation and inhibit apoptosis [50], suggesting that increased *TNF *transcription in BT.siMUC1 could contribute to the increased proliferation seen in these cells.

Interestingly, the increase in *TNF *is accompanied by decreased transcription of *TIMP3*, encoding tissue inhibitor of metalloproteinases (TIMP)3. The TIMP family disrupts the function of matrix metalloproteinases (MMPs), generally resulting in decreased invasion [51]. TIMP3 is unique in that it can also inhibit TNFα converting enzyme (TACE), which activates TNFα by cleaving it from the cell surface [50]. Reduced expression of TIMP3 would, therefore, foster signaling through TNFα by releasing inhibition of TNFα converting enzyme. In agreement with this, TIMP3 can promote apoptosis [52]; thus, its down-regulation in BT.siMUC1 provides another mechanism by which these cells are able to resist the increased apoptosis expected with loss of MUC1.

Another TIMP target responds to MUC1 siRNA, as 468.siMUC1 cells show significantly increased expression of *MMP2 *(encoding MMP-2/gelatinase A), the product of which degrades type IV collagen [52]. In breast cancer, the ratio of active to latent MMP-2 increases with tumor progression; MMP-2 may facilitate both angiogenesis and metastasis [52]. Its increase after loss of MUC1 is, therefore, unexpected, but at least two factors may clarify this result. First, though MMP-2 levels are increased in mouse mammary tumors, its expression is confined to the stroma [53], suggesting that increased *MMP2 *transcription after loss of the epithelium-specific MUC1 might reflect a shift towards a more mesenchymal phenotype. Second, MMP-2 levels are increased by overexpression of erbB2 [52]; previous studies have shown that erbB2 and Muc1 expression are mutually exclusive in mammary tumors [27], implying that *MMP2 *might be part of a transcriptional profile linked to low MUC1 levels.

It is intriguing that, despite increased *MMP2 *transcription, invasion is decreased in 468.siMUC1 cells, even on collagen IV. This may reflect insufficient activation of MMP-2, as the precursor protein must be cleaved for enzymatic function [52]. Alternatively, the slowed invasion of these cells may relate to impaired adhesion resulting from decreased transcription of *ITGAV *and *ITGB1 *(α_v _and β_1 _integrins, respectively). Integrin signaling is tied to life-or-death decisions in epithelial cells, and integrin expression is vital for processes from wound healing to metastasis [54]. Integrin α_v_β_3 _is implicated in facilitating metastasis of breast cancer cells to bone [55]; decreased transcription of *ITGAV *after MUC1 siRNA may, therefore, suggest that MUC1 is involved in this lethal process as well.

The MUC1 oncogene has been linked to apoptosis [18,20,26], proliferation [17], and transcription [21,23-25] in cancer. However, the two cell lines chosen for our study display very different responses to MUC1 siRNA, indicating that regulation of MUC1 in breast cancer is likely quite complex and cautioning against over-generalization of results from individual cell lines. Previous reports suggest that, though most studies outline a clearly oncogenic role for MUC1 in breast cancer, the exact details may vary depending on factors such as cell type and signaling context. For example, MUC1 stimulates Fas-mediated apoptosis in CHO cells [26], quite unlike the inhibition of apoptosis seen in other cell lines. Similarly, though MUC1 drives mammary oncogenesis in its own right [9] and facilitates tumorigenesis driven by other oncogenes [7,8], Muc1 is selectively down-regulated in c-neu-induced mouse mammary tumors [27], indicating that the context of oncogenic signaling is vital to understanding the function of MUC1.

Thus, it is important to consider the relative levels of knockdown of MUC1 in the two cell lines: BT-20 cells reduce MUC1 expression after siRNA less strongly than do MDA-MB-468 (50% versus 75% knockdown, respectively). As MUC1 serves as a scaffold [11], overexpression of MUC1 relative to its associated signaling proteins might create a dilution effect, sequestering signal transducers away from each other; this would be relieved by MUC1 siRNA. Thus, enough MUC1 may be retained in BT.siMUC1 cells for its oncogenic effects, while signaling complex formation would be enhanced by lowering the amount of MUC1 relative to other signaling proteins.

## Conclusion

The contrast between the MDA-MB-468 and BT-20 lines in response to MUC1 siRNA serves as a reminder that simplified models such as cell lines fail to encompass the complexity of intact biological systems. This report describes transcriptional alterations seen after MUC1 knockdown: decreased transcription of *MAP2K1*, *VEGF*, *PDGFA*, *ITGAV*, *TIMP3*, *CDC25A*, and *JUN*, and increased transcription of *MMP2*, *TNF*, and *RAF1*. The alterations in *MAP2K1*, *RAF1*, and *JUN *represent a novel means by which MUC1 can affect ERK1/2 signaling: transcriptional regulation of MAPK pathway members. Oncogenic events are also altered in both cell lines after MUC1 siRNA. These results strengthen the growing ties linking MUC1 and transcriptional regulation, and suggest that the role of MUC1 in breast cancer may be more complex than a direct correlation between MUC1 level and oncogenic function.

## Abbreviations

BrdU = bromodeoxyuridine; EGF = epidermal growth factor; EGFR = epidermal growth factor receptor; ERK = extracellular signal regulated kinase; MAPK = mitogen activated protein kinase; MEK = MAPK and ERK kinase; MMP = matrix metalloproteinases; MUC1-CT = MUC1 cytoplasmic tail; MUC1-EX = MUC1 extracellular subunit; PBS = phosphate-buffered saline; PI = propidium iodide; siRNA = small interfering RNA; TIMP = tissue inhibitor of metalloproteinases; TNF = tumor necrosis factor.

## Competing interests

The authors declare that they have no competing interests.

## Authors' contributions

CLH performed all studies and composed the manuscript. SJG participated in the design and coordination of the studies and contributed strongly to the revision of the manuscript. Both authors have read and approved the manuscript.
